# Screening and Validation of Highly-Efficient Insecticidal Conotoxins from a Transcriptome-Based Dataset of Chinese Tubular Cone Snail

**DOI:** 10.3390/toxins9070214

**Published:** 2017-07-06

**Authors:** Bingmiao Gao, Chao Peng, Bo Lin, Qin Chen, Junqing Zhang, Qiong Shi

**Affiliations:** 1 Hainan Provincial Key Laboratory of Research and Development of Tropical Medicinal Plants, Hainan Medical University, Haikou 571199, China; gaobingmiao1982@163.com; 2 Shenzhen Key Lab of Marine Genomics, Guangdong Provincial Key Lab of Molecular Breeding in Marine Economic Animals, BGI Academy of Marine Sciences, BGI Marine, BGI, Shenzhen 518083, China; pengchao@genomics.cn; 3 Hainan Provincial Key Laboratory of Carcinogenesis and Intervention, Hainan Medical University, Haikou 571199, China; linbo_752@163.com; 4 School of Agricultural and Forestry Science and Technology, Hainan Radio & TV University, Haikou 570028, China; chenqin13141314@163.com

**Keywords:** conotoxin, insecticidal activity, *Conus betulinus*, Chinese tubular cone snail

## Abstract

Most previous studies have focused on analgesic and anti-cancer activities for the conotoxins identified from piscivorous and molluscivorous cone snails, but little attention has been devoted to insecticidal activity of conotoxins from the dominant vermivorous species. As a representative vermivorous cone snail, the Chinese tubular cone snail (*Conus betulinus*) is the dominant *Conus* species inhabiting the South China Sea. We sequenced related venom transcriptomes from *C. betulinus* using both the next-generation sequencing and traditional Sanger sequencing technologies, and a comprehensive library of 215 conotoxin transcripts was constructed. In our current study, six conotoxins with potential insecticidal activity were screened out from our conotoxin library by homologous search with a reported positive control (alpha-conotoxin ImI from *C. imperialis*) as the query. Subsequently, these conotoxins were synthesized by chemical solid-phase and oxidative folding for further insecticidal activity validation, such as MTT assay, insect bioassay and homology modeling. The final results proved insecticidal activities of our achieved six conotoxins from the transcriptome-based dataset. Interestingly, two of them presented a lot of high insecticidal activity, which supports their usefulness for a trial as insecticides in field investigations. In summary, our present work provides a good example for high throughput development of biological insecticides on basis of the accumulated genomic resources.

## 1. Introduction

Cone snails, a large group of carnivorous predators, are usually classified into fish-hunting, snail-hunting and worm-hunting groups [1,2,3]. They are effectively venomous to worms, snails, and fishes by using a deadly combination of paralyzing conotoxins. The fish-hunting group contains the least number of species, while some of them are assessed as deadly to humans. A larger number of species belong to the snail-hunting group that is dangerous by means of their aggressive behavior, although some have been implicated in unconfirmed fatalities. The largest worm-hunting group contains about 80% of the *Conus* genus, while they seem to be nonthreatening [2,3,4].

There are more than 700 *Conus* species in the world, and most of them are distributed throughout tropical and subtropical waters, such as Hainan Island, Philippine Islands, and Pacific Ocean [1]. The venom gland of cone snails is a rich source of bioactive peptides called conotoxins, which have been proved to present many physiological activities [3,4,5]. Each *Conus* species typically contains over 100 conotoxins as potential pharmacological targets; therefore these snails constitute the largest library of natural drug candidates [6]. It has been reported that a total of more than 70,000 conotoxins have been identified from various cone snails around the world [2,7,8]. 

Usually, conotoxins are divided into many different pharmacological families depending on the types of their molecular targets and corresponding physiological activities. They target a wide range of receptors and ion channels [9,10]. Regarding specific targets, some conotoxins have been developed as drug leads for novel analgesics and effective research agents to distinguish subtypes of nervous receptors and ion channels [11,12]. In 2004, ω-conotoxin MVIIA (ziconotide), the most famous conotoxin, became the first marine-derived compound approved by the America FDA for the treatment of pain [13,14].

Previous conotoxin studies are mainly concerned with analgesic and anti-cancer activities, but less attention has been devoted to the potential insecticidal activity for the development of biological pesticides. The Chinese tubular cone snail, *C. betulinus*, is a vermivorous species with a wide distribution in the South China Sea. In our previous study [15], transcriptomes of venom from this *Conus* species were sequenced using both the next-generation sequencing and traditional Sanger sequencing technologies, and a comprehensive library of 215 conotoxin transcripts was constructed. 

**Figure 1:**
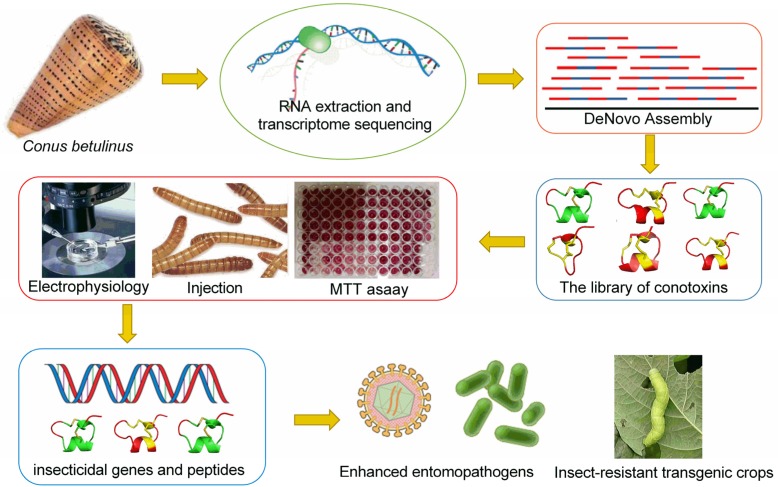
Strategy for high throughput development of bio-pesticides from *Conus* venoms.

However, the functional characteristics of these conotoxins have been rarely reported. There have been only a few reports on the insecticidal activity of peptides, and no highly effective insecticidal conotoxins has been identified. With references from the literature [16], we can screen out certain conotoxins with potential insecticidal activity in our previously established library of peptides using homologous alignment. High insecticidal activity can be subsequently verified through insecticidal bioassays and chemical assays (such as MTT assay). Molecular targets can be further identified by homology modeling and electrophysiology. The main purpose of this work is to screen out conotoxins with high insecticidal activity, which can be used as synergistic genes for construction of recombinant baculoviruses (for subsequent produce of efficient and safe bio-pesticides) or generation of transgenic crops (Figure 1). 

In our present study, conotoxins with potential insecticidal activity were screened out from our constructed library [15] by a homologous search with a reported positive control (alpha-conotoxin ImI from *C. imperialis*) as the query. Subsequently, these conotoxins were synthesized by chemical solid-phase and oxidative folding for further insecticidal activity validation. This is the first report of insecticidal conotoxins in Chinese tubular cone snail.

## 2. Results

### 2.1. Screening out Six Potential Insecticidal Conotoxins for Chemical Synthesis

Alpha-conotoxin ImI (GCCSDPRCAWRC) from *C. imperialis* has been reported to block nAChRs (nicotinic acetylcholine receptors) in *Drosophila melanogaster* [16]. Therefore, it has a potential insecticidal activity. Based on the reported peptide sequence of ImI, we screened the library of conotoxins from *C. betulinus* [15] by using the homologous alignment method. As a result, six homologous conotoxins were obtained, which are in the range of 11 to 22 amino acid residues in length (Table 1). They all contain the representative four cysteines (CC-CC or CC-C-C) for formation of two disulfide bonds, in which loop 1 (between the first and the third cysteines) and loop 2 (between the second and the fourth cysteines) are variable and determine selectivity of corresponding nAChR subtypes [17]. 

For convenience of experiment and recording, we named the six conotoxins as 2-01 to 2-06 respectively (see detailed sequences in Table 1). These conotoxins and the positive control (ImI) were synthesized by chemical solid-phase and oxidative folding (see more details in the section of Materials and Methods) for further insecticidal activity validation, including MTT assay, insect bioassay and homology modeling.

### 2.2. MTT Assay Data

**Figure 2:**
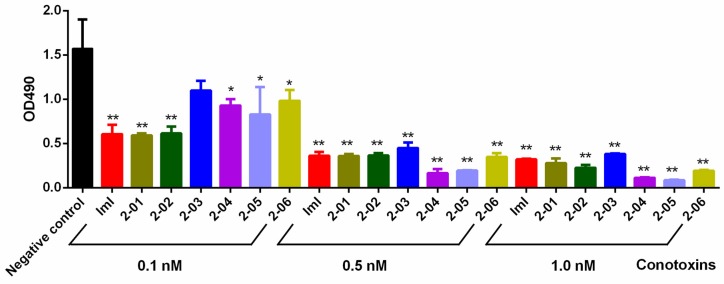
Inhibitory effects of conotoxins on growth of insect sf9 cells. Significance (compared with the negative control): * *p* < 0.05; ** *p* < 0.01.

Inhibitory effects of the six screened conotoxins on growth of insect Sf9 cells were tested by the MTT method [18]. In general, the results (Figure 2) demonstrated significant differences between experimental groups and the negative control (incubation in 0.7% NaCl without conotoxins). The conotoxins 2-01 and 2-02 presented similar inhibitory effects as the positive control (IMI), while 2-03 always showed relatively lower inhibitory effects no matter whether a higher dose or a lower dose of conotoxins (0.1, 0.5 and 1.0 nM of conotoxins in the incubation medium) was applied. Interestingly, the inhibitory effects of 2-04 to 2-6, compared to that of ImI, increased from relatively low (at the incubation dose of 0.1 nM) to relatively high (at the higher dose of 0.5 and 1.0 nM). The *IC*50 value of each conotoxin was calculated to be at the range of 0.13 ~ 0.35 nm (ImI: 0.13 nM, 2-01: 0.13 nM, 2-02: 0.14 nM, 2-03: 0.35 nM, 2-04: 0.18 nM, 2-05: 0.16 nM, 2-06: 0.22 nM) by Graphpad Prism (version 6.0, Graphpad Software Inc., La Jolla, CA, USA).

### 2.3. Insect Bioassay Data

**Figure 3:**
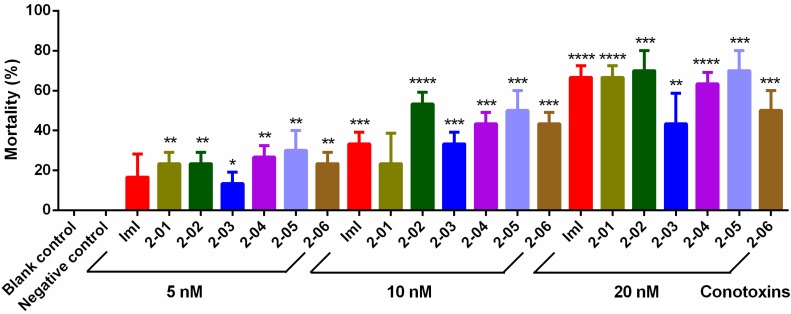
Insecticidal effects of conotoxins at different concentrations. Columns represent the mean ± SEM for three replicates of 10 insects for each dose. Significance (compared with the negative control): * *p* < 0.05; ** *p* < 0.01; *** *p* < 0.001, **** *p* < 0.0001.

The six screened conotoxins and two controls were directly injected into the lower abdomen of mealworms to evaluate their insecticidal effects [19]. Mortality of mealworms in the blank group (the blank control without injection) and the negative control (injection of 0.7% saline) was 0%, while the mortality in the conotoxin groups was significantly increased when compared to the two controls, indicating that the injection method was feasible to evaluate the insecticidal activity of conotoxins. The final results (Figure 3) proved the insecticidal activities of our achieved six conotoxins from the transcriptome-based dataset. Generally speaking, with elevation of the injection dose, the six conotoxins along with the positive control (ImI) presented an elevation of effect in killing mealworms. Interestingly, 2-02 and 2-05 always showed relatively higher insecticidal effects, no matter whether a higher dose or a lower dose of conotoxins (injection of 5, 10 and 20 nM of conotoxins) was applied. The *LC*50 value of each conotoxin was calculated by Graphpad Prism to be in the range of 11.7 ~ 18.5 nM (ImI: 15.0 nM, 2-01: 15.6 nM, 2-02: 12.1 nM, 2-03: 22.5 nM, 2-04: 14.0 nM, 2-05: 11.7 nM, 2-06: 18.5 nM).

In combination with the MTT assay data, we observed good inhibition on sf9 cells and killing effects on mealworms by 2-01, 2-02, 2-04 and 2-05, and two of them (2-02 and 2-05) are always the strongest with a mortality rate of insects of ~70% in the high-dose (20 nM) groups (Figure 3).

### 2.4. Prediction of 3D Structures with Homology Modeling 

**Figure 4:**
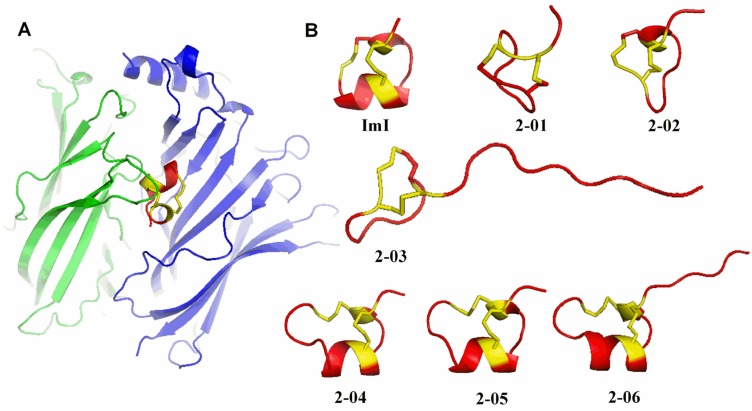
Predicted structures of the six achieved conotoxins on basis of homology modeling. (**A**) A homology model of the conotoxin (red) binding with a nAChR at the interface between 2 subunits (α3β2; in green and blue colors); (**B**) Stereoview of each conotoxin structure. Note that every conotoxin has two disulfide bridges (yellow) arranged in the pattern of Cys1–Cys3 (loop 1) and Cys2–Cys4 (loop 2).

Based on the reported structure of ImI (PDB: P50983), we predicted 3D structures of the achieved six conotoxins (Figure 4) by using homology modeling [20]. Similar to ImI, these predicted structures are stabilized by two intrachain disulfide bridges with the representative pattern of Cys1–Cys3 and Cys2–Cys4 (Figure 4A). As a α-conotoxin, ImI performs actions by binding to acetylcholine receptors, where the number and attributes of amino acids between loop 1 (between the first and third cysteines) and loop 2 (between the second and the fourth cysteines) are variable and determine selectivity of the corresponding nAChR subtypes [17,21]. Since the N-terminal sequences and length affect the biological activity of peptides [22], we propose similar insecticidal activity among four achieved conotoxins (2-01, 2-02, 2-04 and 2-05) and ImI, based on their similar structures (Figure 4B). Nevertheless, the N-terminal sequences of conotoxins 2-03 and 2-06 are so long to reduce the insecticidal activity, which are consistent with the MTT assay data and insect bioassay data. Therefore, these predicted structures by homology modeling are instructive for functional prediction and validation. This seems to be a good method for low-cost and highly-efficient screening of potential candidates before performance of expensive experimental validation.

## 3. Discussion

Insect pests are a major cause for worldwide reduction of crop yields. Each year insect pests cause serious damage in the agricultural field that costs billions of dollars annually to farmers. Chemical insecticides have been developed to be the dominant method to control pest populations [23]; however, insects are developing resistance to many chemical pesticides and some popular pesticides may have potentially harmful effects or risks on the environment and human health. These problems urge us to develop novel and safe insecticidal compounds or peptides for effective pest control [23,24]. 

Insect baculoviruses are known to regulate many insect populations in nature. However, their host-specificity is very high, usually restricted to even a single or a few closely related insect species. To date, baculovirus insecticides have been widely applied to control insect pests in agriculture and forestry in China and Latin America [23,25]. In 2014, 57 products from 11 viruses were authorized as commercial viral insecticides by China Ministry of Agriculture [25]. Despite several cases of successful viral insecticide use, insecticide failures have also been frequently documented. The main problems are related to their relatively slow speed of action, low virulence against older insect instars, and ultraviolet radiation sensitivity. Subsequently, genetic modification has been used to improve and overcome such issues [23,25]. 

Insect predators and parasites use venoms to immobilize their prey. Fortunately, venoms of these molluscivorous cone snails are in fact a mixture of toxins that may have a narrow spectrum of activity against various insects, hence it is possible to isolate toxin genes that target insects with high specificity. Most of the known *Conus* species specialize in hunting worms; however, previous conotoxin studies are mainly concerned with molluscivorous and piscivorous species. Much less attention has been devoted to in-depth examination of insecticidal activity to develop conotoxins into biological pesticides [26,27]. Some vermivorous *Conus* species are among the largest conoideans, such as *C. betulinus*, which has been observed by divers to eat large polychaetes [2,28]. 

Peptides are a valuable alternative to chemical pesticides for control of insect pests in agriculture because of their high killing efficiency, low pest-specificity and human safety [5,24,29]. Reported examples of insecticidal conotoxins include the α-conotoxin ImI from *C. imperialis* [16], omega conotoxin GVIA from *C. geographus*, MVIIC from *C. magus*, and kappa conotoxin k-PVIIA from *C. purpurascen* [30,31]. Conotoxins in the venom of cone snails can specifically bind to the Na^+^, K^+^ or Ca^2+^ ion channels and nAChRs in the insect nerve membranes; nontoxicity or very weak toxicity to mammals and crustaceans, however, can specifically paralyze and poison insects [32]. Because it has been proven that conotoxins are usually more selective for insect nAChRs, they will be safer in the field pest management practices [33]. Our preliminary electrophysiological experiments also validated that some conotoxins had little binding activity against acetylcholine receptors of humans. For example, a mucher high dose (10 μm) of the conotoxin 2-05 could block only 41.24% of the observed ACh-induced current for human nAChR α3β4. However, the *IC*50 and *LC*50 values of 2-05 to insect cells/insects reveled in the present study are 0.16 nM and 11.7 nM respectively (Figure 2 and Figure 3). The remarkable differences confirmed that these conotoxins seem to be specific for paralysis and poisoning to insects, while they presented only a weak toxicity to mammals. 

Genetic engineering has been applied to modify the insecticidal activity of baculoviruses, and presents a great elevation to improve the effectiveness of insect viruses [34]. The main purpose of realizing genetic modification of wild-type baculoviruses by inserting toxin fragment(s) is to increase the effectiveness and speed to kill target insect pests. It was reported that a mite neurotoxin gene was cloned into an AcMNPV for expressing toxins to paralyze a host [35]. Consistent with this observation, some insecticidal venom peptides from cone snails, scorpions or spiders were inserted into the wild baculoviruses for protection of cotton, poplar and tobacco [26,36,37]. 

## 4. Conclusions

In our previous report [15], a comprehensive library of conotoxins (215 conotoxin transcripts) was constructed on the basis of the transcriptomes of venom for *C. betulinus*. However, little information is available about the functional characteristics of these conotoxins. As *C. betulinus* is hunting worms, these conotoxins are supposed to have certain insecticidal activity. Our present work provides the first report to develop biological insecticides in a high throughput way. Conotoxins with potential insecticidal activity were screened out by a homologous search from our previously constructed conotoxin library. After chemical synthesis and functional validation, six conotoxins with insecticidal activities were obtained, in which two (2-02 and 2-05) presented a high insecticidal activity. Their *IC*50 values are 0.14 nM and 0.16 nM respectively, and their corresponding *LC*50 values are 12.1 nM and 11.7 nM respectively. The results prove that our current method is effective for screening and obtaining conotoxins that target insects with high specificity. Our present work also provides a good example for high throughput development of potential biological insecticides on basis of the accumulated genomic resources.

## 5. Materials and Methods

### 5.1. Screening of Insecticidal Conotoxins

ImI was reported to have insecticidal activity [16]. Based on this sequence (KJ801971.1; Table 1), homologous alignment was performed to screen peptides with potential insecticidal activity from our previously constructed library of conotoxins for *C. betulinus* [15]. These sequences were aligned for comparison by using ClustalX 2.1 and GeneDoc Multiple Sequence Alignment Editor and Shading Utility version 2.7.000 [38]. To represent identical or homologous residues in each sequence, amino acids were shaded in different colors (Table 1). 

### 5.2. Solid-Phase Peptide Synthesis

The screened conotoxins were chemically synthesized by a solid-phase method [11] at GL Biochem Ltd. (Shanghai, China). As reported previously [17], we dissolved each crude conotoxin peptide in 30% acetonitrile for separation by RP-HPLC (Waters, Milford, MA, USA) on a Vydac C18 column (Waters, Milford, MA, USA), which was equilibrated in Solution A (30% acetonitrile, 0.1% TFA). Subsequent purification was performed using a linear gradient (30 to 60%) of Solution B (90% acetonitrile, 10% H_2_0, 0.1% TFA) for 30 min, at a flow rate of 1 mL/min. Each conotoxin at a final concentration of 0.1 mg/mL was folded overnight (for formation of stable disulfide bridges) in an Optimized Buffer at room temperature. To obtain high-quality folded peptides (up to 98% homogeneity), we applied linear acetonitrile gradient (15–30%) over 40 min in the final RP-HPLC purification step. ESI-MS mass spectrometry (Shimadzu, Kyoto, Japan) was employed to verify the mass identity between synthetic and folded conotoxins.

### 5.3. MTT Assay

At a common density of 5 × 10^3^ cells/well in 100 µL of culture medium (with or without the synthetic conotoxins), Sf9 cells were seeded in 96-well microtiter plates (Costar, High Wycombe, Bucks, UK) for incubation at 27 °C for 48 h. Ten microliters of 3-(4,5-dimethylthiazol-2-yl)-2,5- diphenyltetrazolium bromide (MTT reagent) was added to each well and incubated for 4 more hours. Subsequently, after removal of the MTT solution and addition of 100 μL of DMSO (dimethyl sulfoxide), the plates were incubated for 15 min. Absorbance of DMSO extracts were measured at 490 nm using an enzyme immunoassay analyzer (BIO-RAD, Hercules, CA, USA). 

### 5.4. Insecticidal Bioassay

Mealworms between the 3rd and 4th instar (at an average of 180 mg/individual) were collected. Chicken feed, purchased from a local company, was provided ad libitum as mealworms’ food. The synthesized conotoxins were dissolved freshly in 0.7% saline to the final desired concentration (25 nM, 50 nM or 100 nM) immediately before experiments. Injections of conotoxins or negative control (0.7% saline alone) were performed using a liquid micro-injector (Shanghai High Pigeon, Shanghai, China) in a volume of 5 μL, followed by 15 μL of 0.7% saline to wash any residues in the injector. Three replicates of 10 mealworms were used for each conotoxin concentration or control. At 48 h after injection, the mealworms were checked to determine behavioral changes and mortality. 

All animal experiments were approved by the Institutional Review Board on Bioethics and Biosafety of BGI (No. FT15103) on 6 July, 2015.

### 5.5. Homology Modeling

All the modelling, docking and simulations were performed in Discovery Studio Client 4.0 (Accelrys, San Diego, CA, USA). The molecular models of extracellular ligand-binding domains of the human nAChRs (such as α3β2) were generated based on the template of Ac-AChBP structure using the homology modelling program Modeler version 9.0 [39]. The structure of α-conotoxin ImI (PDB: P50983) was downloaded from the public PDB database. Conotoxin docking was based on the reference model of the Ac-AChBP/GIC complex [19]. The models were refined with a side-chain refinement and energy minimization process. All modelling and docking structures were verified by the program Profiles-3D in the Discovery Studio platform, as well as by the MolProbity server [20]. All structural figures were generated with PyMol (version 1.7.0.0, DeLano Scientific, San Carlos, CA, USA).

### 5.6. Statistical Analysis

All data are presented as the mean ± SEM. Student’s *t* test were performed for statistical analyses by using Graphpad Prism (version 6.0, Graphpad Software Inc., La Jolla, CA, USA). 

## Figures and Tables

**Table 1:** Comparison of the six achieved conotoxins with the positive control (ImI).

Name	Conotoxin Sequence	Accession No.	Mw-ca	Mw-ms
ImI	---------GCCSDPR--CAWR----C*--	KJ801971.1	1356.6	1355.634
2-01	--------SECCIRN-FLC-------C*--	KU317668.1	1290.6	1289.715
2-02	-----R---PCCPRDTW-C-------CI--	KU564001.1	1452.8	1452.708
2-03	TLQMLRGVQICCPYILW-C-------CLIP	KU563944.1	2567.3	2566.937
2-04	------G--GCCSHPA--CGVNHPELC*--	KU563886.1	1683.9	1682.828
2-05	------G--GCCSYPP--CIASNPK-CG--	KU564009.1	1657.0	1656.768
2-06	--AISSG--ACCAYPP--CFEAYPERCL--	KU563887.1	2351.8	2351.677

Note: Conserved residues (with similar properties) among different conotoxins are highlighted in the same background color; * amidated C-terminus; Mw-ca, calculated molecular weight; Mw-ms, MS measured molecular weight.
